# Joint contractures in severe burn patients with early rehabilitation intervention in one of the largest burn intensive care unit in China: a descriptive analysis

**DOI:** 10.1186/s41038-019-0151-6

**Published:** 2019-05-20

**Authors:** Jianglin Tan, Jian Chen, Junyi Zhou, Huapei Song, Huan Deng, Ming Ao, Gaoxing Luo, Jun Wu

**Affiliations:** 1 Institute of Burn Research, State Key Laboratory of Trauma, Burns and Combined Injuries, Chongqing Key Laboratory for Disease Proteomics, Southwest Hospital, Third Military (Army) Medical University, Chongqing, 400038 China; 2 Department of Burns, First Affiliated Hospital of Sun Yat-Sen University, Guangzhou, 510080 China

**Keywords:** Burn, Rehabilitation, Contracture, Intensive care unit

## Abstract

**Background:**

Joint contracture is the major clinical complication in burn patients, especially, the severe burn patients. This study aimed to investigate the number and severity of joint contractures in patients with burns affecting greater than or equal to 50% of the total body surface area (TBSA) undergoing early rehabilitation in a burn intensive care unit (BICU).

**Methods:**

We analyzed burn patients with burns affecting greater than or equal to 50% of the TBSA admitted to a BICU who received early rehabilitation within 7 days post-injury from January 2011 to December 2015. Demographic and medical information was collected. The range of motion (ROM) of different joints was measured 1 month post-admission. Spearman’s correlation coefficient and logistic regression analysis was used to determine predictors of the presence and severity of contractures.

**Result:**

The average affected TBSA of the included burn patients was 67.4%, and the average length of stay in the BICU was 46.2 ± 28.8 days. One hundred and one of 108 burn patients (93.5%) developed at least one joint contracture. The ROM in 67.9% of the affected joints was mildly limited. The majority of contractures in severe burn patients were mild (37.7%) or moderate (33.2%). The wrist was the most commonly affected joint (18.2%), followed by the shoulder, ankle, hip, knee, and elbow. A predictor of the presence of contractures was the length of hospital stay (*p* = 0.049). The severe contracture was related to the area of full-thickness burns, the strict bed rest time, and the duration of rehabilitation in BICU. The length of rehabilitation stay (days) in patients with moderate contracture is 54.5% longer than that in severe contracture (*p* = 0.024)

**Conclusion:**

During the long stay in BICU, the length of rehabilitation stay in a BICU could decrease the severity of contractures from severe to moderate in the patients with equal to 50% of the TBSA. Hence, this research reveals the important role of early rehabilitation interventions in severe burn patients.

**Electronic supplementary material:**

The online version of this article (10.1186/s41038-019-0151-6) contains supplementary material, which is available to authorized users.

## Background

The survival rates in severe burn patients have been significantly improved in the past few decades [1, 2]. As a result, the attention to burn care is shifting from saving life to issues of rehabilitation, such as function and social integration. During rehabilitation for burn patients, contracture is the major clinical complication in those with deep dermal and full-thickness burns that can induce the loss of joint mobility and dysfunction in ambulation and transfers, fine motor tasks, and activities of daily living [3–5].

In burn patients, injury severity demographics, scar contractures, and rehabilitation treatment time are related to patient outcomes [6]. The acute development of burn scar contracture in burn survivors who receive adequate care and rehabilitation while in the hospital can be avoided [7]. The factors contributing to joint contracture can occur in two stages. In acute stage, burn patients receiving long-term immobilization of their extremities by positioning, pain management, and splinting were easily to have joint contracture [8, 9]. During the wound healing stage, the high-risk factors included race, gender, age, burn site, multiple surgical procedures, healing time, and burn severity of scar formation which could result in high prevalence rate of joint contracture [10–12]. Thus, there is a high prevalence of joint contracture in burn patients. The use of a rotating bed is the standard method for preventing pressure damage to the skin or skin grafts during the acute period in mainland China; however, this technique usually leads to immobilization for a long time. Traditional rehabilitation in mainland China may begin at least 1 month after injury when most of the wounds were healed [13]. Therefore, how to balance immobilization and early rehabilitation in a burn intensive care unit (BICU) [14] is still a challenge.

In the literature, Goverman et al. retrospectively reviewed 1865 burn patients and found that 33% of these patients developed at least one contracture at discharge [15]. Gangemi et al. reported contracture in 31% of 220 burn patients, among which 8% patients needed surgery [16]. Schneider et al investigated 11 burn patients and found that the mean number of joint contractures in an 11 patient group was four [17]. However, there was no investigation concerning both the frequency and severity of contractures in burn patients with burns affecting greater than or equal to 50% of the total body surface area (TBSA) undergoing early rehabilitation in a BICU. What are the effects of early rehabilitation in severe burn patients? Therefore, we collected demographic and medical data and analyzed the incidence, severity, and frequency of joint contracture in burn patients. Moreover, we also studied the factors that related to the incidence and severity of joint contractures.

## Methods

### Setting

As one of the largest burn units in mainland China, the Institute of Burn Research of the First Affiliated Hospital of Third Military Medical University (Army Medical University) has 125 inpatient beds, including 20 beds in the BICU, and has more than 1500 burn inpatients per year. Two rehabilitation therapists have provided early physical and occupational therapies in this BICU since 2011.

When the residual wound areas of severe burn patients are less than 5–10% of the TBSA, the patients have stable vital signs including blood pressure, blood oxygen saturation, pulse rate, respiratory rate, and body temperature, and there is no need for life support; these severe burn patients are transferred from the BICU to the mild burn ward. In the burn ward, patients receive standard burn care and are discharged from the hospital when there are no residual wound areas and there is no need for inpatient treatment.

### Data collection

Clinical data of selected burn patients admitted to the BICU from January 2011 to December 2015 were extracted from case records and analyzed. This retrospective study was approved by the Ethical Committee in the First Affiliated Hospital of Third Military Medical University (Army Medical University). All the patients’ information was kept confidential. The data included demographic information (age and sex), burn injury characteristics, length of BICU stay (BICU LOS), length of hospital stay (LOS)= BICU LOS+ mild ward LOS, inhalation injury, ventilator-dependent days, strict bed rest time, and BICU rehabilitation days (= BICU LOS − interrupted by surgery LOS). Severe burn patients in the BICU who needed more than moderate help to turn or reposition their bodies were transferred to a turning bed or a suspension bed. And the nurses or therapists helped them turn or reposition every 2 h. These details were recorded in their case records, and the length of time was defined as strict bed rest time in this study. The active range of motion (ROM) of different joints was measured using a goniometer and an inclinometer with a standardized technique approximately 1 month post-admission to the BICU. Joint muscle action in each plane was assigned a normal ROM based on physical examination conventions. Each impaired joint muscle action was assigned a severity rating. These ratings were determined by dividing the normal active ROM value equally in thirds (mild, moderate, and severe; Additional file 1: Table S1). A limitation in the ROM in at least one plane of motion at a specified joint was considered to be contracture at that joint. The most severe active ROM deficit was considered the category of joint contracture.

The inclusion criteria were as follows: (1) 16–65 years old, (2) admission to the BICU within 7 days post-injury, (3) early rehabilitation therapies performed within 7 days post-injury from therapists in the BICU from January 2011 to December 2015, (4) burn size equal to or more than 50% of the TBSA, and (5) survival from the burn injury.

The exclusion criteria were as follows: (1) same BICU LOS as the LOS (indicating that the patient might not meet the criteria for being transferred out of the BICU for various reasons, such as death), (2) lack of assessment data (indicating that the patients transferred to other hospitals or departments without first monthly rehabilitation assessment or the patients refused to receive rehabilitation treatment because of financial problem on hospital expense), (3) no rehabilitation performed, and (4) preexisting physical disabilities.

### Early rehabilitation protocol in the BICU

**Fig. 1:**
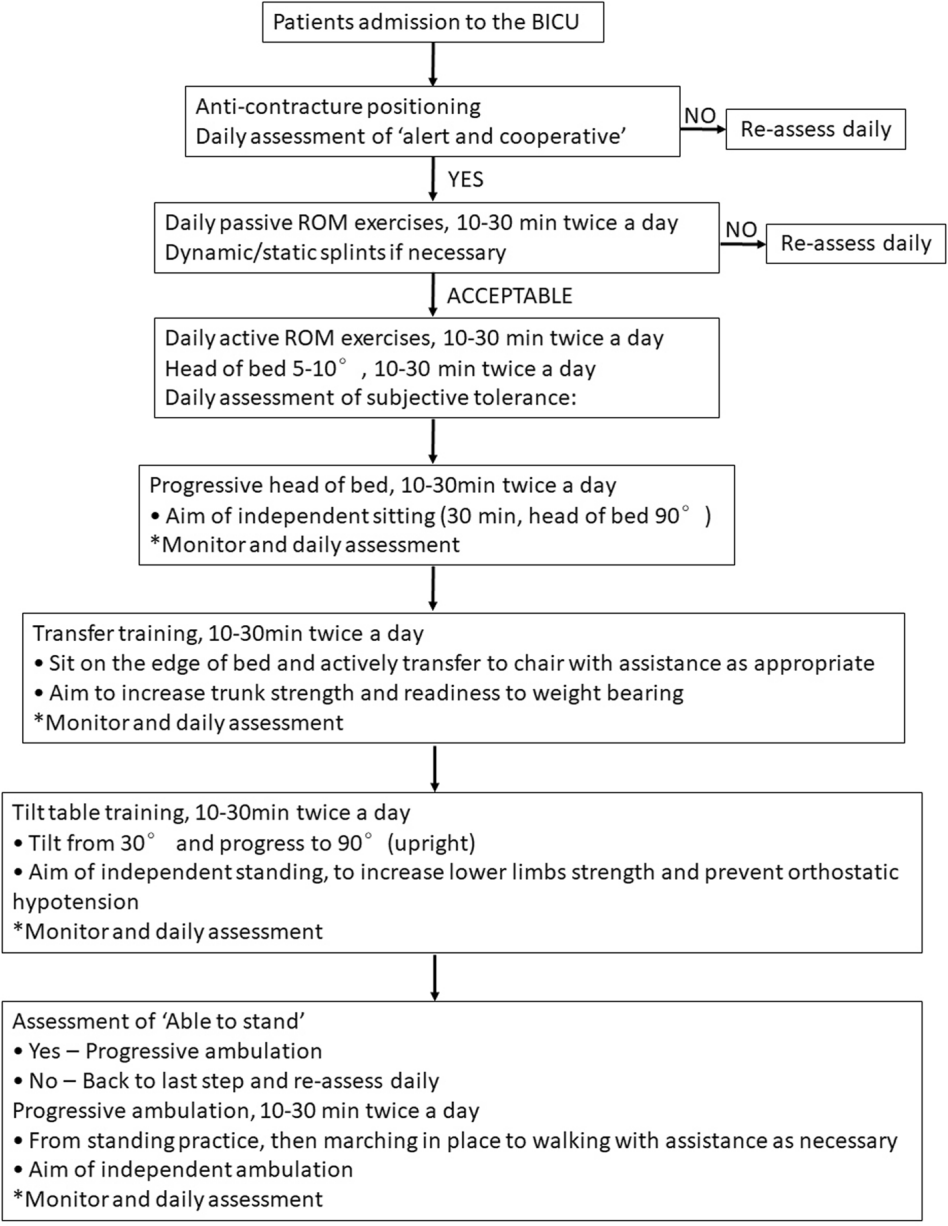
Flow chart of early rehabilitation in the burn intensive burn unit (BICU). The severe burn patients receive routine rehabilitation provided by rehabilitation therapists. Asterisk denotes monitor and daily assessment: monitor the changes in heart rate, respiratory rate, and blood pressure and daily assess the subjective tolerance. No significant changes and acceptable tolerance: progress; if no: back to the last step and re-assess daily. *ROM* range of motion

Early rehabilitation in this manuscript was defined as the rehabilitation management provided by therapists on burn patients within 7 days post-injury. An initial assessment for patients in the BICU was performed within 7 days. Routine early rehabilitation intervention was provided by rehabilitation therapists after the shock period. The patients were assisted to attain an anticontracture position, followed by daily passive ROM exercises and dynamic/static splints used in an antideformity position if necessary. Active ROM exercises, transfer training, and tilt table training were performed under the rehabilitation therapists’ professional guidance. If the patients were able to stand (including patients with mechanical ventilation), the therapists performed progressive ambulation around the bed under monitoring the patients’ vital signs, including changes in heart rate, respiratory rate, and blood pressure, and performed a daily assessment of whether the patients could tolerate these rehabilitation exercises. Rehabilitation progressed based on the patient’s medical condition and a monthly assessment report. The rehabilitation protocol for severe burn patients in the BICU is explained in Fig. 1.

During the acute burn surgery, the rehabilitation management on surgical area would be interrupted for a few days. Therefore, the BICU rehabilitation days were also decreased. The surgical area was dressed with thick gauze in functional position after surgery. The therapists would monitor and assess the surgical area, followed by the doctors. In general, the rehabilitation management would restart at 3–5 days in split-thickness skin graft and 5–7 days in full-thickness skin graft after surgery.

### Statistical analysis

The data were expressed as the mean ± standard deviation (SD) and median [Q1–Q3]. SPSS 23.0 software (Chicago, IL, U.S.) was used for statistical analysis in this study. Because most of the data were not normally distributed, nonparametric correlation (Spearman’s correlation) analysis was first used to determine the relations to the presence, severity, and number of contractures. Then, we used binary logistic regression to analyze the predictors of the presence of contractures. For the three-level multinomial model in the severity of contractures (mild contracture, moderate contracture, and severe contracture), we tended to use ordinal logistic regression but it violates the proportional odds assumption. So, we chose a multinomial regression model instead given its less binding assumptions. Statistically significant was considered as *p* < 0.05.

## Results

### Demographic and injury-related medical data

During the study period from January 2011 to December 2015, 286 patients (≥ 50% of the TBSA affected) were collected in the BICU. Among these patients, a total of 108 patients met the inclusion criteria, while 178 burn patients were excluded. In the excluded patients, there were 37 dead patients, 34 patients younger than 16 years, 29 patients older than 65 years, 19 admission patients without 7 days post-injury, and 59 patients transferred to other hospitals or other departments with less than 20 days BICU stay. These excluded patients lack the monthly rehabilitation assessment. The demographic and medical data are presented in Table 1.

**Table 1:** Demographic and medical characteristics of the population collected in the burn intensive care unit (BICU)

Groups	Excluded patients					Included patients
	Dead patients	Patients younger than 16 years	Patients older than 65 years	Admission patients without 7 days postinjury	Patients with less than 20 days BICU stay	
Number of patients	37	34	29	19	59	
Total number of patients			178			108
Male (%)	89.2%	44.1%	55.2%	70.0%	67.8%	79.6%
Female (%)	10.8%	55.9%	45.8%	30.0%	32.2%	20.4%
Age at injury, (mean ± SD, years)	36.9 ± 16.5	6.1 ± 4.6	71.2 ± 3.6	25.3 ± 8.5	43.3 ± 12.8	37.3 ± 14.1
High school or above (%)	92.6%	16.1%	17.2%	62.1%	74.1%	88.9%
Length of stay, (median [Q1-Q3], days)	16.0 [6.0–25.0]	37.0 [16.0–61.0]	4.0 [1.5–6.5]	42.0 [15.0–73.0]	9.5 [5.5–15.0]	83.5 [52.0–133.3]
Length of BICU stay, (mean ± SD, days) or (median [Q1-Q3], days)	16.0 [6.0–25.0]	15.0 [4.0–27.0]	4.0 [1.5–6.5]	14.5 ± 6.6	9.8 ± 5.5	46.2 ± 28.8
TBSA burned, (mean ± SD, %)	86.3 ± 10.6	70.0 ± 14.5	62.1 ± 10.3	56.9 ± 6.9	78.9 ± 15.7	67.4 ± 12.2
Deep thickness burned, (mean ± SD, %)	26.6 ± 23.7	31.0 ± 13.4	23.0 ± 18.1	14.2 ± 8.8	32.6 ± 19.9	36.4 ± 20.1
Full-thickness burned, (mean ± SD, %)	57.6 ± 23.7	40.0 ± 25.5	39.3 ± 23.3	12.0 ± 8.3	44.3 ± 25.8	34.1 ± 22.3
Etiology (%)						
Scald	8.1%	45.2%	0	20.0%	15.2%	15.3%
Fire/flame	48.6%	54.8%	89.7%	40.0%	54.2%	70.3%
Chemical agent	21.2%	0	0	20.0%	6.8%	3.6%
Blast/explosion	21.2%	0	10.3%	0	15.3%	6.3%
Electricity	0	0	0	0	5.1%	1.8%
Other	0	0	0	20%	3.4%	2.7%
Inhalation injury (%)	63.6%	32.3%	37.8%	10.0%	30.6%	47.7%
Mild	12.1%	8.8%	17.2%	10.0%	5.1%	20.7%
Moderate	27.3%	17.6%	10.3%	0	15.3%	17.1%
Severe	24.2%	5.9%	10.3%	0	10.2%	9.9%
Ventilator-dependent patients (%)	40.5%	8.8%	13.8%	0	20.3%	37.8%
Ventilator-dependent days, (median [Q1-Q3], days)	12.0 [5.0–22.0]	5.0 [2.0–10.0]	3.0 [1.0–5.0]	0	6.0 [3.0–11.0]	5.0 [3.0–10.0]
Strict bed rest time, (mean ± SD, days)	17.5 ± 13.8	17.9 ± 7.0	6.8 ± 2.9	4.5. ± 2.6	9.3 ± 5.3	34.6 ± 27.9
BICU rehabilitation (days)	7.8 ± 4.2	14.7 ± 4.9	2.3 ± 1.2	13.3 ± 6.2	5.7 ± 2.3	29.7 ± 6.3

*SD* standard deviation, *TBSA* total body surface area

There were 86 males and 22 females in the included patients. The average age was 37.3 years, and the mean affected TBSA was 67.4% (67.4 ± 12.2). The average deep thickness burned was 36.4% (36.4 ± 20.1). The full-thickness burned was 34.1% (34.1 ± 22.3). The median length of hospital stay was 83.5 days (83.5 (52.0–133.3)), and the average BICU LOS was 46.2 days (46.2 ± 28.8). In this study, fire/flame was the most common cause of burns, accounting for 70.3% of the etiologies. Among the 108 enrolled patients, 47.7% experienced inhalation injury (mostly the mild inhalation injury (20.7%)), and 37.8% of the subjects were supported by ventilators. The median duration of ventilator dependency was 5 days (5.0-10.0). The average duration of strict bed rest was 34.6 days (34.6 ± 27.9), and the average time of BICU rehabilitation was 29.7 days (29.7 ± 6.3).

In the excluded patients, there were five sub-groups including dead patients, patients younger than 16 years old, patients older than 65 years old, admission patients without 7 days post-injury, and patients with less than 20 days BICU stay. There were 37 dead patients in the excluded group with an average age of 36.9 years old. The TBSA burned area (86.3% ± 10.6) and full-thickness burned area (57.6% ± 23.7) in dead patients group were all much higher than other four excluded sub-groups and the included group. Similarly, the percent of severe inhalation injury (24.2%) and ventilator-dependent patients (40.5%) were much higher in the dead group. The length of BICU rehabilitation in the excluded group including the five sub-groups (7.8 ± 4.2, 14.7 ± 4.9, 2.3 ± 1.2, and 13.3 ± 6.2) was less than those in the included group (29.7 ± 6.3). Interestingly, the incidence of blast/explosion (21.2%) increased in the dead group. The fire/flame was also the first injury factor. But the incidence of scald (45.2%) increased in patients younger than 16 years old.

### Severity and frequency of joint contracture

In the 108 surveys, the ROM of a total of 2436 joints was limited, and the ROM was mildly limited in 67.9% of joints (Table 2). The wrist was the most commonly affected joint (18.2%), and the elbow was the least affected joint (15.0%) (Table 3). One hundred and one of 108 burn patients showed at least one joint contracture. The total number of joint contractures was 525. There were 69 patients (63.9%) suffering from six joint contractures affecting the shoulder, elbow, wrist, hip, knee, and ankle (Table 4).

**Table 2:** Severity and frequency of active range of motion (ROM) limitation by joint muscle action

Joint	Muscle action	Severity						Total
		Mild		Moderate		Severe		
		Left	Right	Left	Right	Left	Right	
Shoulder, n(%)	Flexion	30(18.9)	34(21.4)	31(19.5)	35(22.0)	17(10.7)	12(7.5)	159
	Extension	39(41.9)	46(49.5)	6(6.5)	2(2.2)	0(0)	0(0)	93
	Abduction	25(16.0)	35(22.4)	46(29.5)	43(27.6)	4(2.6)	3(1.9)	156
	Horizontal abduction	40(45.5)	40(45.5)	1 (1.1)	5(5.7)	3(3.4)	2 (2.3)	88
	Horizontal adduction	44(34.6)	46(36.2)	15(11.8)	18(14.2)	3(2.4)	1(0.8)	127
Elbow, n(%)	Flexion	55(30.9)	62(34.8)	22(12.4)	25(14.0)	10(5.6)	4(2.2)	178
	Extension	72(48.3)	70(47.0)	2(1.3)	5(3.4)	0(0)	0(0)	149
Wrist, n(%)	Flexion	47(27.5)	54(31.6)	30(17.5)	25(14.6)	9(5.3)	6(3.5)	171
	Extension	49(28.7)	48(28.1)	26(15.2)	27(15.8)	11(6.4)	10(5.8)	171
Hip, n(%)	Flexion	41(24.3)	50(29.6)	29(17.2)	20(11.8)	14(8.3)	15(8.9)	169
	Extension	27(42.2)	27(42.2)	1(1.6)	1(1.6)	4(6.3)	4(6.3)	64
	Abduction	60(42.3)	63(44.4)	13(9.2)	12(8.5)	4(2.8)	3(2.1)	142
	adduction	40(31.7)	38(30.2)	19(15.1)	16(12.7)	4(3.2)	9(7.1)	126
Knee, n(%)	Flexion	49(30.6)	47(29.4)	16(10.0)	22(13.8)	9(5.6)	17(10.6)	160
	Extension	68(50.7)	62(46.3)	0(0)	3(2.2)	1(0.7)	0(0)	134
Ankle, n(%)	Dorsiflexion	47(28.3)	45(27.1)	7(4.2)	9(5.4)	30(18.1)	28(16.9)	166
	Plantar flexion	77(46.1)	78(46.7)	5(3.0)	4(2.4)	3(1.8)	0(0)	167
Total, n(%)		810(33.3)	845(34.7)	269(11.0)	272(11.2)	126(5.2)	114(4.7)	2436
		1655(67.9)		541(21.8)		240(9.9)

**Table 3:** Contracture severity and frequency by joint in patients with burns affecting greater than or equal to 50% total body surface area undergoing early rehabilitation in burn intensive care unit

Joint	Contracture severity			Total, n(%)
	Mild	Moderate	Severe	
Shoulder	23	40	24	87 (17.0)
Elbow	33	33	11	77 (15.0)
Wrist	27	39	27	93 (18.2)
Hip	35	21	29	85 (16.6)
Knee	37	24	23	84 (16.4)
Ankle	38	13	35	86 (16.8)
Total, n(%)	193 (37.7)	170 (33.2)	149 (29.1)	512

**Table 4:** Contracture frequency in patients with burns affecting greater than or equal to 50% total body surface area undergoing early rehabilitation in burn intensive care unit

Patients with at least one contracture	101 (*N* = 108)
Total number of contractures	525
Average number of contractures per person, mean±SD	4.86 + 1.81
Patients number with two contractures	6
Patients number with three contractures	12
Patients number with four contractures	7
Patients nemuber with five contractures	7
Patients number with six contractures	69

*SD* standard deviation

### Risk factors for joint contracture

Spearman’s correlation coefficient analysis predicted that there was no relationship between the presence of contracture and either the demographic data (age and sex), affected TBSA, burn injury characteristics, BICU LOS, inhalation injury, or duration of ventilator dependency, or strict bed rest. However, the length of hospital stay was related to the presence of contractures (*p* = 0.035) (Table 5). Then, binary logistic regression analysis indicated the same result that the length of hospital stay was positively related to the presence of contractures (*p* = 0.049) (Table 6). Full-thickness burns area and the duration of strict bed rest showed a negative association with contracture severity. However, rehabilitation time (days) in the BICU was associated with a reduction of severe burn contracture (*p* = 0.024). The full-thickness burn is 81.9% more likely to be associated with a severe rather than mild contracture (*p* = 0.024). Similarly, the full thickness is 56.7% associated with a severe rather than mild contracture (*p* = 0.020). The strict bed rest time (days) is 76.8% associated with severe contracture compared to mild contracture (*p* = 0.012). However, the amount of rehabilitation stay (days) was 54.5% more likely associated with a moderate contracture than a severe contracture (*P* = 0.024) (Table 7).

**Table 5:** Univariate analyses with Spearman’s correlation coefficient of relations of the presence of contractures

Variable	*ρ*	*P* value
Age	− 0.164	0.090
Gender	− 0.040	0.683
TBSA (%)	− 0.145	0.136
Deep thickness	− 0.066	0.499
Full thickness	− 0.018	0.855
Severity of inhalation injury	− 0.047	0.628
Time post-burn (hour)	0.123	0.204
LOS (days)	0.203	0.035*
Classification of etiology	0.124	0.200
Ventilator dependent days	− 0.112	0.249
Strict bed rest time	0.042	0.668
BICU LOS (days)	0.074	0.445
Time post-admission of inpatient rehabilitation	− 0.053	0.584
Number of surgery	− 0.026	0.792
BICU rehabilitation (days)	0.040	0.682

*BICU LOS* length of burn intensive care unit stay, *TBSA* total body surface area, *LOS* length of hospital stay

*Means *p* < 0.05

**Table 6:** Binary logistic regression analysis of predictors of the presence of contractures

Variable	*B*	Odds ratio	*P* value	95% confidence interval
Age	− 0.207	0.931	0.056	0.865–1.002
TBSA (%)	6.851	0.939	0.069	0.877–1.005
LOS (days)	0.072	1.022	0.049*	1.000–1.045
Strict bed rest time (days)	− 0.063	0.961	0.528	0.848–1.088
BICU LOS (days)	0.022	1.292	0.212	0.864–1.930
Number of surgery	8.446	0.775	0.444	0.404–1.488
BICU rehabilitation (days)	− 0.207	0.813	0.268	0.564–1.172

*BICU LOS* length of burn intensive care unit stay, *TBSA* total body surface area

*Means *p* < 0.05

**Table 7:** Multinomial logistic regression analysis of predictors of the severity of contractures

	Mild contracture				Moderate contracture			
Variable	*B*	Odds ratio	*P* value	95% confidence interval	*B*	Odds ratio	*P* value	95% confidence interval
Full thickness	−1.709	0.181	0.024*	0.041–0.802	− 0.837	0.433	0.020*	0.215–0.874
Strict bed rest time (days)	−1.461	0.232	0.012*	0.074–0.724	0.124	1.132	0.652	0.660–1.942
BICU LOS (days)	0.509	1.663	0.341	0.584–4.733	0.843	2.323	0.061	0.961–5.616
BICU rehabilitation (days)	− 0.148	0.862	0.768	0.322–2.309	0.606	1.545	0.024^*^	1.322–1.923
Number of surgery	0.331	1.394	0.341	0.703–2.766	− 0.105	0.900	0.482	0.672–1.207

*BICU LOS* length of burn intensive care unit stay

Reference category: severe contracture

*Means *p* < 0.05

## Discussion

In this study, a total of 286 burn patients with ≥ 50% of the TBSA were admitted to BICU. The percentage of male (55.2–89.2%) was much higher than female (10.8–45.8%) in all groups except the excluded patients younger than 16 years old group (male vs female: 44.1% vs 55.9%). The percentage of male burn patients was similar to Goverman et al.’s 2016 study (77.5%) on the adult contractures in burn injury [15]. In addition, according to our early survey on the current status of burn rehabilitation services in China, early rehabilitation therapy is still not widely accepted in the hospital, especially in the acute burn care stage. Only 29.2% of burn centers began early rehabilitation therapy in 1 week after burn [13]. Therefore, the burn patients transferred to the primary hospital or other departments could not receive rehabilitation therapy. Interestingly, the excluded dead patients group had a much higher ratio of chemical agent and blast/explosion than the included group. These patients in this excluded dead group were easily to suffer from chemical poison and combined injuries, such as kidney failure, severe inhalation injury, or heart injury, inducing multiple organ failure and death. Therefore, the severe inhalation injury percentage of inhalation injury, the rate of ventilator-dependent patients, and ventilator-dependent duration in the excluded dead group were all much higher compared to other excluded sub-groups and the included group.

Larger TBSA burned, being more likely to cross multiple joints, is a high risk of developing contractures. Therefore, how to decrease the morbidity of contracture and the severity of contracture in larger TBSA burned patients is very important in the burn treatment. In our study, many factors contributed to burn contracture in severe larger burn patients. Interestingly, risk factors such as TBSA burns and medical problems that have demonstrated as predictors of the presence of contractures [15] had no relationship with the occurrence of contracture. Our results indicated that the longer the stay in the hospital was, the more likely burn patients were to have contractures. This may be because the longer time the patients spent in the hospital, the more severe burns they had. Thus, these patients had a high presence of contractures. Moreover, the incidence of contracture in severe burn patients (93.5%) was significantly higher than that in general burn patients (38–54%). The depth of the burn, duration of strict bed rest, and duration of BICU rehabilitation analyzed by logistic regression were shown to be associated with the severity of contracture in our study. The main column of interest in Table 7 is headed “odds ratio.” An odds ratio of 1 is a null value, meaning that the predictor has no effect on the outcome of interest. But an odds ratio greater or less than 1 is associated with an increase or decrease in the odds of the outcome of interest. In our study, the odds ratio associated with full thickness in mild contracture is 0.181, meaning that the full thickness is 81.9% (= 1–0.181) more likely to be associated with a severe rather than mild contracture. The full thickness is 56.7% (= 1–0.433) associated with a severe rather than mild contracture. The odd ratio associated with strict bed rest time is 0.232, meaning that the strict bed rest time (days) is 76.8% (= 1–0.232) associated with severe contracture rather than mild contracture. As another result, the odd ratio associated with BICU rehabilitation days is 1.545, meaning that the BICU rehabilitation days in moderate contracture are 54.5% (=1.545–1) longer than that in severe contracture. Therefore, the full-thickness depth, duration strict bed rest, and BICU rehabilitation days had higher values contributing to the severity of contracture joints. Furthermore, moderate burn patients received longer rehabilitation interventions than severe burn patients. It indicated that the duration of rehabilitation interventions could decrease the severity of contractures. Okhovatian et al. reported that burn patients who underwent long-term and early rehabilitation had fewer contractures than those in a control group [18]. Similarly, our data indicated that early rehabilitation could be useful for severe burn patients.

In inclusion population of our study, only seven of 108 enrolled burn patients developed no joint contracture; thus, 93.5% of severe burn patients had at least one contracture at discharge. The seven burn patients without joint contractures have the same characteristics. First, the affected TBSA of the burn patients was 50–55%. Second, the burns at the joint location in these patients were superficial partial thickness burns; thus, the burns at the joint could heal in 2 weeks without scar formation. With early rehabilitation, these seven burn patients were transferred from a rotating bed to a normal hospital bed in a short time, leading to effective limb movement, especially active ROM. The severe burn patients had an obviously higher incidence of joint contracture (93.5%) than the general burn patients (38–54%) at hospital discharge [15, 19]. This may in part be because burns affecting a larger TBSA were likely to cross more joints; therefore, these burns were associated with at a statistically higher risk of joint contracture. In this report, our data showed that the upper extremities had a higher incidence of joint contracture than the lower extremities, which is similar to the results in the general population of burn patients [19, 20]. In terms of etiology, fire/flame was the main cause of burns in our study (70.3%). The patients were likely to try to put out the fire with their hands in an unconscious response. As a result, the wrist was the most severely and frequently involved joint (18.2%).

Martin’s work indicated that postural treatment, kinesiotherapy, and functional recovery were the three pillars of rehabilitation in extensively burned patients [21]. Porter’s study showed that structured rehabilitative exercise was a safe and efficacious strategy to restore physical function in burn patients [22]. The rehabilitation of burn patients requires a coordinated multidisciplinary team that includes burn surgeons and nurses, physical and occupational therapists, and rehabilitation nurses [23, 24]. In this study, rehabilitation assessment was performed early by the therapists during the postshock phase after admission to the BICU, immediately followed by a comprehensive rehabilitation program developed by the professional therapists and nurses. Appropriate positioning, maintenance of ROM, and splint support were the main methods of rehabilitation used to keep the joints in noncontracted and functional positions in patients with unstable vital signs. To minimize the incidence of joint contracture resulting from long-term immobilization, passive and active ROM exercise and mobility training performed at least twice a day were added to the rehabilitation plan for severe burn patients after the shock phase in this study.

As a developing country, China has a higher incidence of burns than developed countries [13, 25]. In mainland China, burn patients with burns affecting greater than or equal to 50% of the TBSA are classified as the most serious cases and are directly admitted to the BICU. Some severe burn patients are immobilized on a rotating bed after the early shock phase [26]. The rotating beds used in BICU only allow positioning of the patient in a supine or a prone position, which might restrict joint movement and lead to joint contracture. Usually, traditional rehabilitation interventions in mainland China are provided after most wound areas are healed. In contrast, early rehabilitation in burn patients showed improvement in joint ROM and a shorter BICU LOS and in the hospital [27–29]. According to the analysis in this study, we found that when early rehabilitation was performed within 7 days post-injury, severe burn patients could have fewer contractures of decreased severity. Hence, the data in this study could help to investigate the epidemiology of joint contractures in severe burn patients, to identify the associated risk developing joint contracture, and to either prevent or decrease the severity of joint contracture by interventions including early rehabilitation in the BICU during the early stages.

### Limitations

Rehabilitation is an individualized treatment for each severe burn patient. It is difficult to develop a standard therapy and control group (this is also not an ethical issue). There were limitations in our study because we only analyzed patients who underwent early rehabilitation in our burn center compared with those who did not undergo early rehabilitation in the literature. In this study, we have 178 excluded patients without rehabilitation data which may cause bias in the data analysis. It is better to collect all the rehabilitation data to compare the excluded patients with included patients in the future study. The excluded patients with low incomes prefer to refuse rehabilitation treatment or transfer to the primary hospital without rehabilitation because of hospital expense. This was also a risk factor for the data bias. Hence, the data here may not be representative of all burn patients and other burn centers. In addition, the data in this study only included the BICU LOS instead of the LOS in the mild burn wards, which may not represent the entire process of contracture progression in burn patients. Moreover, another limitation in our study was that we did not explore the ROM of the metacarpophalangeal and interphalangeal joints, which might affect the fine motor movements of the fingers. Future research may focus on the effect of early rehabilitation on the movement of small joints.

Okhovatian’s study reports that physiotherapy treatment for 30–45 min two to three times per day in burn patients on the first day after admission was associated with 6% contractures compared with 73% contractures in those who underwent 15–20 min of rehabilitation once a day approximately 2 weeks after their general condition [18]. Thus, analysis of the relationship between contractures and the duration of rehabilitation (minutes and frequency) in severe patients may have more significance. Another shortcoming was that we did not analyze the duration of rehabilitation in minutes.

## Conclusion

Our data in this study showed that most severe burn patients with burns affecting greater than or equal to 50% of the TBSA exhibited joint contracture in the BICU. The length of rehabilitation stay in a BICU could decrease the severity of contractures from severe to moderate. Hence, this research reveals the important role of early rehabilitation interventions in severe burn patients.

## Additional file

Additional file 1: **Table S1.** Range of motion severity ratings by joint muscle action. (PDF 125 kb)

## Abbreviations

BICU: Burn intensive care unit
LOS: Length of hospital stay
ROM: Range of motion
TBSA: Total body surface area
