# Supplementary material for: Joint contractures in severe burn patients with early rehabilitation intervention in one of the largest burn intensive care unit in China: a descriptive analysis
Source: Burns Trauma. 2019 May 20;7:17. doi: 10.1186/s41038-019-0151-6 (PMC6526598; doi:10.1186/s41038-019-0151-6)
Supplement: Supplementary file 1 — Table S1. Range of motion severity ratings by joint muscle action. (PDF 125 kb) [file 41038_2019_151_MOESM1_ESM.pdf]

Additional file 1: Table S1: Range of motion severity ratings by joint muscle action

Joint	Muscle Action	Contracture Deverity		
		Mild	Moderate	Severe
shoulder	Flexion	120-180	60-119	＜60
	Extension	32-50	16-31	＜16
	Abduction	120-180	60-119	＜60
	Horizontal Abduction	40-26	25-13	＜13
	Horizontal Adduction	135-90	89-45	＜45
Elbow	Flexion	93-140	46-92	＜46
	Extension	-20-0	-40--21	＜-40
Wrist	Flexion	80-53	26-52	＜26
	Extension	47-70	23-46	＜23
Hip	Flexion	80-120	40-79	＜40
	Extension	20-30	10-19	＜10
	Abduction	26-40	13-25	＜13
	Adduction	24-38	12-23	＜12
Knee	Flexion	100-150	50-99	＜50
	Extension	-20-0	-40--20	＜-40
Ankle	Dorsiflexion	14-20	7-13	＜7
	Plantar flexion	32-50	16-31	＜16
